# Hemorheological and microcirculatory effects of Pentaglobin therapy in an experimental model of fulminant sepsis

**DOI:** 10.3389/fphys.2026.1816549

**Published:** 2026-04-24

**Authors:** Adam Attila Matrai, Balazs Ujhelyi, Adam Varga, Sandor Richard Zahorszki, David Martin Adorjan, Zoltan Toth, Norbert Nemeth, Bela Fulesdi, Adam Deak

**Affiliations:** 1 Department of Operative Techniques and Surgical Research, Faculty of Medicine, University of Debrecen, Debrecen, Hungary; 2 Department of Anesthesiology and Intensive Care, Faculty of Medicine, University of Debrecen, Debrecen, Hungary; 3 Department of Medical Microbiology, Faculty of Medicine, University of Debrecen, Debrecen, Hungary

**Keywords:** fulminant sepsis, hemorheology, microcirculation, Pentaglobin, red blood cell aggregation, red blood cell deformability

## Abstract

**Introduction:**

Sepsis is a life-threatening syndrome characterized by dysregulated immune response to infection and multi-organ dysfunction. One of the key components of this condition is microcirculatory dysfunction. The aim of our study was to evaluate the effects of IgM-enriched immunoglobulin (Pentaglobin, PG) on microcirculation and hemorheological parameters in a porcine model of fulminant sepsis induced by intravenous Escherichia coli (*E. coli*) suspension.

**Methods:**

Thirty female juvenile pigs were randomized into four groups: Control, *E. coli* bacteremia, *E. coli* + PG parallel, and *E. coli* + delayed PG. Under anesthesia, the external jugular veins and the right femoral artery were cannulated. The Control group received fluid therapy only. In all sepsis groups, 38 ml of *E. coli* suspension was administered intravenously over 3 hours. The *E. coli* + PG parallel group received a 0.75 g/kg Pentaglobin bolus infusion simultaneously with *E. coli*. The *E. coli* + delayed PG group received 0.67 g/kg bolus Pentaglobin 1 hour after sepsis induction, followed by a 0.02 g/kg/h maintenance infusion for 4 hours. Microcirculatory assessments were performed before infusion and every 2 hours until hour 6. Hematological parameters, red blood cell (RBC) aggregation, and blood/plasma viscosity were measured.

**Results:**

The microvascular flow index reached its highest values in the *E. coli* + delayed PG group at hour 6 (neck: 2.65 ± 0.23; sublingual: 2.67 ± 0.38). Perfused vessel density was highest in the Control group (sublingual: 1.58 ± 0.39 mm/mm2). Microcirculatory values were worse in the *E. coli* bacteremia group, indicating marked edema, RBC aggregates, and vascular heterogeneity. *E. coli* + PG parallel, and *E. coli* + delayed PG groups showed reduced hematocrit values (p<0.001 vs. baseline) and less pronounced increases in whole blood viscosity and RBC aggregation compared to the *E. coli* bacteremia group.

**Discussion:**

Fulminant sepsis resulted in severe microcirculatory and hemodynamic disturbances. Pentaglobin therapy mitigated the decline in tissue perfusion, attenuated the elevation of blood viscosity and red blood cell aggregation.

## 1 Introduction

Fulminant sepsis caused by Escherichia coli (*E. coli*) is a rapidly progressing and life-threatening condition characterized by an uncontrolled immune response to infection, leading to severe systemic inflammation, coagulopathy, hypoperfusion, tissue hypoxia, endothelial dysfunction, and multiple organ failure (Trappe and Riess, 2005; Singer et al., 2016; Gyawali et al., 2019; Giustozzi et al., 2021; Jarczak et al., 2021; Kuwabara et al., 2022). *E. coli* is one of the most common and serious pathogens causing sepsis, often causing urinary tract and intra-abdominal infections, which can later spread systemically throughout the body. Fulminant sepsis progresses rapidly, so early recognition and timely initiation of aggressive treatment are very important, including immediate antibiotic therapy targeting Gram-negative bacteria, fluid replacement, and supportive care to mitigate organ failure. Despite all this, mortality remains high, especially in septic shock, where the mortality rate can reach 60% (Jarczak et al., 2021). The severe clinical picture associated with *E. coli* sepsis and the rapidly deteriorating health status emphasize the importance of early diagnosis and urgent, targeted treatment (Gyawali et al., 2019; Jarczak et al., 2021). Hospital mortality caused by this condition is a significant public health problem worldwide (Csomós et al., 2005; Kadri et al., 2017; Kópházi et al., 2023).

In sepsis, a cytokine storm plays a central role in the development of organ dysfunction. Levels of proinflammatory cytokines such as IL-6, IL-8, and TNF-α rise; these cytokines are associated with the clinical symptoms of sepsis and with the SOFA (Sequential Organ Failure Assessment) scoring system, which is used to evaluate organ dysfunction (Bozza et al., 2007; You, 2025). As sepsis progresses, the body enters an immunosuppressive state as a compensatory mechanism. Production of pro-inflammatory cytokines decreases, while anti-inflammatory cytokines, including IL-10, become predominant. Although IL-10 reduces inflammation, persistently high levels lead to immunosuppression, which may increase the risk of nosocomial infections and mortality (Gupta et al., 2022; Cao et al., 2023; Vella et al., 2025; Wang et al., 2025).

Hemorheological and microcirculatory disorders may play a central role in the development of organ failure. During sepsis, functional capillary density decreases, and blood flow becomes heterogeneous, with reduced perfusion in some capillaries and normal flow in others. This heterogeneous blood flow can lead to localized tissue hypoxia despite normal systemic oxygen supply (Vincent and De Backer, 2005; De Backer et al., 2014; Miranda et al., 2016; Raia and Zafrani, 2022). Endothelial dysfunction impairs vasomotor regulation, increases vascular permeability, and enhances leukocyte and platelet adhesion, which can further impair microvascular flow (Vincent and De Backer, 2005; De Backer et al., 2014; Raia and Zafrani, 2022). The shape-changing ability of red blood cells may change, limiting their passage through narrow capillaries, and their tendency to aggregate may increase, which may increase blood viscosity, thereby impairing microcirculation and oxygen transport (Vincent and De Backer, 2005; De Backer et al., 2014; Nader et al., 2019). Impaired microcirculation can lead to tissue hypoxia and organ dysfunction even when systemic hemodynamics appear to be normal (Vincent and De Backer, 2005; Miranda et al., 2016; Gruartmoner et al., 2017; Raia and Zafrani, 2022).

Microcirculatory disturbance can be exacerbated by increased whole blood viscosity and increased red blood cell aggregation, which increase resistance and impair capillary perfusion, promoting the development of hypoxic tissue areas alongside adequately oxygenated areas, which is characteristic of microcirculatory dysfunction in sepsis (Vincent and De Backer, 2005; De Backer et al., 2014; Nader et al., 2019). The severity and persistence of microcirculatory and hemorheological abnormalities may be closely related to the extent of organ failure and mortality in septic patients. Despite advances in systemic hemodynamic management, targeted treatment of microcirculatory dysfunction remains challenging, and it is important to evaluate the effects of therapies on microcirculation and red blood cell rheology (Vincent and De Backer, 2005; De Backer et al., 2014; Miranda et al., 2016; Gruartmoner et al., 2017).

The microcirculation comprises vessels with a diameter below 100 µm, including arterioles, capillaries, venules, and arteriovenous anastomoses. Declining tissue perfusion and hypoxia induce the release of vasodilators such as nitric oxide (NO) in an attempt to maintain microvascular blood flow. When compensatory mechanisms are exhausted, excessive NO diffuses across the capillary wall, inhibit mitochondrial cytochrome-c oxidase, and - while reducing cellular oxygen demand - ultimately contributes to membrane injury and necrosis.

Increased capillary permeability in sepsis promotes interstitial edema and enlarges intercapillary distances, further impairing oxygen and nutrient delivery. Concurrent activation of the coagulation system leads to microthrombus formation and progressive deterioration of tissue perfusion. Microcirculatory assessment, therefore, relies both on the evaluation of tissue substrate delivery and uptake and on direct microvascular imaging.

Pentaglobin, an IgM-enriched immunoglobulin preparation, may have therapeutic potential in sepsis due to its immunomodulatory effects (balancing the dysregulated immune response, anti-inflammatory action, pathogen neutralization and immune recalibration) and may also have a beneficial effect on the inflammatory response and microcirculatory function. Pentaglobin may promote microvascular recruitment and improve tissue perfusion during sepsis primarily through its immunomodulatory effects, as these effects indirectly restore microcirculatory function (Domizi et al., 2019). Improved microcirculatory flow may reduce tissue hypoxia and organ dysfunction, which are the main drivers of sepsis mortality. The IgM-rich preparation can be considered as an adjunctive therapy to antibiotics and hemodynamic support, aimed at restoring immune balance and improving outcomes. The immunomodulatory properties of Pentaglobin help regulate the excessive inflammatory response in sepsis, while its positive effects on micro-circulatory function can improve tissue perfusion and oxygen supply. These combined effects may contribute to its therapeutic potential in the treatment of sepsis, particularly through the treatment of microcirculatory dysfunction underlying organ failure in septic patients (Cui et al., 2019; Domizi et al., 2019).

We aimed to investigate the hemorheological and microcirculatory changes occurring in *E. coli*-induced fulminant sepsis, as well as the differences between administering Pentaglobin at different times on the aforementioned parameters, which may contribute to the optimization of therapeutic strategies used in sepsis. Data on the potential effects of IgM-rich immunoglobulin on the micro-rheology and microcirculation in *E. coli*-induced sepsis are limited. Pentaglobin may improve microvascular perfusion and reduce inflammation through its immunomodulatory effects, as indicated by clinical and experimental studies describing improvements in microcirculatory parameters and cytokine modulation during Pentaglobin treatment (Domizi et al., 2019; Yavuz and Bindal, 2022). However, we have no information regarding the use of Pentaglobin therapy there are any differences in micro-rheological and microcirculatory parameters between Pentaglobin therapy initiated as a preventive measure (e.g., prior to urological, abdominal, and gynecological procedures) and Pentaglobin treatment administered during established sepsis, or whether Pentaglobin administered preventively is capable of limiting sepsis.

## 2 Materials and methods

### 2.1 Experimental animals and protocol

The experiment was conducted in accordance with European Union Directive (63/2010 EU Directive) and Hungarian animal protection law (Act XXVIII of 1998 on the protection and welfare of animals), and with the approval of the University of Debrecen Committee of Animal Welfare (permission registration number: 18/2023/UDCAW). We ensured ventilation (15–20 air changes per hour, sudden fluctuations in humidity were avoided) and heating (central and underfloor heating) in the animal housing facilities, at a temperature of 22–26 °C, appropriate to the body weight of the animals. The animals were fed with a diet appropriate for their species, and drinking water was provided by a self-watering system.

The experiment was performed under general anesthesia. Azaperone was used as premedication (2 mg/kg i.m.; Stresnil, Elanco GmbH, Cuxhaven, Germany), followed by induction with a combination of 15 mg/kg intramuscular ketamine (CP-Ketamine hydrochloride 10%) and 1 mg/kg xylazine (CP-Xylazine hydrochloride 2%). Maintenance anesthesia was performed intravenously with 1 mg/kg xylazine and 10 mg/kg ketamine. Following induction, endotracheal intubation was performed, followed by pressure-supported mechanical ventilation (Aeonmed Respiratory Ventilator VG70, Beijing Aeonmed Co., Ltd., China). The aim of mechanical ventilation was to maintain an arterial partial oxygen pressure of 100 (± 10) mmHg and partial carbon dioxide pressure around 40 (± 5) mmHg. Following surgical exposure of the external jugular veins on both sides and the right femoral artery, we inserted arterial (Getinge PiCCO Catheter 4F, 16 cm, Pulsion Medical System SE, Feldkirchen, Germany) and venous cannulae (Certofix Duo 7F, 20 cm, B. Braun Trading Ltd., Budapest, Hungary) for blood sampling, hemodynamic measurements, and treatment. A suprapubic cystostomy catheter was used to measure hourly urine output. In accordance with clinical practice, volume replacement was used to alleviate the hypovolemic state that develops during sepsis.

In the Control (n=6), *E. coli* bacteremia (n=8), *E. coli* + Pentaglobin (PG) parallel (n=8), and *E. coli* + delayed PG (n=8) groups, pigs received intravenous physiological saline solution (Baxter Sodium Chloride 0.9%, pH=4.5-7, osmolarity: 308 mOsm/l, Baxter Hungary Ltd.) at a rate of 10 ml/kg/hour. In the *E. coli* bacteremia, *E. coli* + PG parallel, and *E. coli* + delayed PG groups, sepsis was induced in the same way: *E. coli* (2.5×10^5^/ml; ATCC 25922, Department of Medical Microbiology, Faculty of Medicine, University of Debrecen) culture was dissolved in physiological saline solution and administered by continuous intravenous infusion according to the following schedule: 2 ml in the first 30 minutes, 4 ml in the next 30 minutes, and then the remaining 32 ml of bacterial culture was administered to the pigs over 120 minutes. A total dose of 9.5×10^6^ *E. coli* was administered to the animals over a period of 180 minutes. The Control group received only volume replacement therapy, whereas animals of the other three groups received *E. coli* suspension. In the *E. coli* bacteriemia, *E. coli* + parallel PG and *E. coli* + delayed PG groups, sepsis induction was identical in all experimental animals. *E. coli* bacteriemia group only received *E. coli* suspension. *E. coli* parallel PG group received IgM-enriched immunoglobulin concomitantly with *E. coli* suspension. *E. coli* delayed PG group administration of Pentaglobin was started 60 min after the start of the *E. coli* suspension.

In the *E. coli* + PG parallel group, 0.75 g/kg body weight of IgM-enriched immunoglobulin (Pentaglobin^®^, Biotest Pharma GmbH, Dreieich, Germany) was administered in a 20-minute bolus infusion simultaneously with the *E. coli* infusion. In the *E. coli* + delayed PG group, starting 60 minutes after the initiation of the *E. coli* infusion, we administered a bolus infusion of 0.67 g/kg body weight, followed by a maintenance infusion of 0.02 g/kg/hour for 240 minutes in the form of Pentaglobin. The amount of immunoglobulin infusion was subtracted from the amount of maintenance saline solution, so that the total volume corresponded to a dose of 10 ml/kg/hour.

At the end of the experiment, the pigs were euthanized under deep anesthesia. During the study, no other drug (including antibiotic treatment) or therapeutic interventions were performed other than intravenous analgesic sedation, physiological saline solution, *E. coli* suspension, immunoglobulin, and pressure-supported ventilation.

### 2.2 Hematological variables measurements

A Sysmex K-4500 microcell counter (TOA Medical Electronics Co., Ltd., Kobe, Japan) was used to measure qualitative and quantitative hematological variables. The following parameters were assessed in this study: red blood cell count (RBC [T/L]), white blood cell count (WBC [G/L]), hemoglobin concentration (Hgb [g/dl]), hematocrit (Hct [%]), mean corpuscular volume (MCV [fl]), mean corpuscular hemoglobin (MCH [pg]), mean corpuscular hemoglobin concentration (MCHC [g/dl]), and platelet count (Plt [G/L]).

### 2.3 Determining red blood cell aggregation

Red blood cell aggregation index values were measured using a Myrenne MA-1 erythrocyte aggregometer (Myrenne GmbH, Roetgen, Germany). This technique uses light-transmittance photometry and requires about 20 µl of whole blood. After disaggregation via a controlled shearing system (shear rate: 600 s^–1^), light transmission was measured for 5 or 10 seconds under static conditions (M values, shear rate: 0 s^–1^) or at a low shear rate (M1 values, shear rate: 3 s^–1^) (Baskurt et al., 2009; Vayá et al., 2009). Measurements were performed at room temperature (20–25 °C). Higher index values (M 5 s, M1–5 s, M 10 s, M1–10 s) indicate greater RBC aggregation (Lee et al., 2007; Vayá et al., 2009).

### 2.4 Testing whole blood and plasma viscosity

Whole blood viscosity (WBV) and plasma viscosity (PV) were determined using a DVNext cone/plate rheometer with cone spindle CPA-40Z (AMETEK Brookfield, Middleborough, Massachusetts, United States) at 37 °C. This technique requires 500 µl of whole blood or plasma. When determining blood viscosity, values corrected for a hematocrit of 40% were also calculated using the mathematical formula recommended by Mátrai et al (Matrai et al., 1987).

### 2.5 Microcirculatory measurements

Non-invasive, direct visualization of microcirculation can be achieved using videomicroscopy. Orthogonal polarization spectral (OPS) imaging employs polarized light and detects depolarized light scattered by moving erythrocytes. Sidestream dark-field (SDF) imaging utilizes a ring of LEDs surrounding a central microscope to minimize surface reflections and improve signal quality. Incident dark-field (IDF) imaging, a refinement of SDF, delivers illumination centrally, enabling higher resolution and contrast (Hutchings et al., 2016).

Videomicroscopic assessments of the microcirculation were performed at predefined time points according to the experimental protocol. Using the CytoCam-IDF device, image sequences were recorded from the skin of the neck and ear, as well as from the sublingual mucosa. Microvascular structures were visualized based on the presence of hemoglobin-containing erythrocytes; consequently, only vessels containing moving red blood cells were detectable. The device illuminates the tissue with green light at a wavelength of 540 nm. Fluorescent light is absorbed by erythrocytes and reflected by surrounding tissues, after which the reflected signal is captured by the CytoCam-IDF’s 14-megapixel camera and converted into digital images (Hutchings et al., 2016).

The videos were analyzed using automated, validated analysis software (CytoCamTools V3 Bedside Manager, Braedius Medical, Huizen, The Netherlands) that provides consistent microvascular parameters, including perfused vascular density (PVD) - the density of the vascular network multiplied by the proportion of perfused vessels - the proportion of perfused vessels (PPV), and the De Backer score. To visually characterize blood flow in the vessels, we used the MFI, or microvascular flow index, which is a dimensionless number.

### 2.6 Statistical analysis

We used GraphPad Prism version 9.1.2 (GraphPad Software, San Diego, California, USA) for statistical analyses. Data are presented as mean ± S.D. (standard deviation). The sample size was estimated using the G*Power statistical software. The normality of the data distribution was tested using the Kolmogorov-Smirnov test. Depending on the test results, differences within groups were analyzed using repeated-measures ANOVA, followed by Dunnett’s multiple comparison test or the Friedman test; differences between groups were analyzed using the t-test for independent samples/Mann-Whitney U test or one-way ANOVA, followed by Tukey’s multiple comparison test or the Kruskal-Wallis test. The level of significance was set at p < 0.05.

## 3 Results

### 3.1 Hematological variables

Hematological analysis revealed significant intergroup differences (**Table 1**). No significant differences were observed in the Control group. In both *E. coli* bacteremia and *E. coli* + PG parallel, *E. coli* + delayed PG groups, the WBC count initially decreased, then increased, with a more pronounced rise at 6 hours in the *E. coli* + PG parallel, and *E. coli* + delayed PG groups, suggesting an immunomodulatory effect of the therapy (*E. coli* + PG parallel 360 min: p=0.0014 vs. base; *E. coli* + delayed PG 240 min: p=0.0016 vs. Control, p=0.0076 vs. base). Red blood cell count, hemoglobin, and hematocrit showed similar trends: slight increases in the *E. coli* bacteriemia group, but decreasing dynamics in the *E. coli* + PG parallel and *E. coli* + delayed PG groups, possibly indicating therapy-related physiological redistribution affecting microcirculation and blood cell dynamics. The platelet count decreased in each group, with a significant decrease in the *E. coli* + delayed PG group compared to the base value (p=0.0443 vs. 120 min, p=0.0026 vs. 240 min, p=0.0051 vs. 360 min).

**Table 1:** Hematological variables in the Control, *E. coli* bacteremia, *E. coli* + PG parallel and *E. coli* + delayed PG groups.

Variables		Control	*E. coli* bacteremia	*E. coli* + PG parallel	*E. coli* + delayed PG
WBC [10^9^/L]	base	11.91 ± 1.51	14.14 ± 2.9	12.67 ± 2.17	13.84 ± 2.95
	120 min	14.06 ± 3.37	13.36 ± 3.39	10.1 ± 1.91*#$	12.72 ± 3.77
	240 min	12.75 ± 1.33	17.01 ± 3.42*	12.77 ± 2.11#	16.45 ± 3.28*$
	360 min	16.22 ± 2.66$	17.56 ± 3.21	15.38 ± 1.18$	16.89 ± 2.94
RBC [10^12^/L]	base	5.86 ± 0.69	5.45 ± 0.67	6.08 ± 1.12	5.16 ± 0.49*
	120 min	5.78 ± 0.75	5.65 ± 0.81	4.8 ± 0.55*#$	5.1 ± 0.87
	240 min	5.66 ± 0.74	5.98 ± 1.47	4.94 ± 0.57$	4.73 ± 0.63*#$
	360 min	5.59 ± 0.53	5.76 ± 0.6	5.24 ± 0.71$	4.8 ± 0.86*#
Hgb [g/L]	base	108.75 ± 16.66	101.44 ± 7.94	102.64 ± 9.02	96.59 ± 8.82
	120 min	103.92 ± 13.34	101.76 ± 13.97	86.93 ± 7.61*#$	91.21 ± 11.99$
	240 min	98.4 ± 6.29	102.14 ± 10.91	86.44 ± 10.79*#$	84.94 ± 13.85*#$
	360 min	102.92 ± 9.89	102.22 ± 9.95	91.69 ± 13.07$	80.88 ± 12.54*#$
Hct [%]	base	35.54 ± 5.66	31.72 ± 4.21	33.3 ± 4.72	30.43 ± 3.22*
	120 min	34.42 ± 4.24	33.12 ± 4.57	27.87 ± 3.34*#$	29.96 ± 3.69*
	240 min	32.56 ± 2.04	33.66 ± 3.3	28.74 ± 3.53*#$	27.97 ± 4.22*#$
	360 min	33.79 ± 3.07	33.68 ± 3.17	30.63 ± 4.48	26.99 ± 3.95*#$
MCV [fL]	base	60.43 ± 3.25	58.18 ± 2.6	57.63 ± 1.54	58.88 ± 1.6
	120 min	60.74 ± 2.91	58.73 ± 2.39$	58.04 ± 1.69*$	59.27 ± 2.11
	240 min	60.69 ± 2.92	58.77 ± 2.42$	58.14 ± 1.66$	59.06 ± 2.01
	360 min	60.53 ± 2.96	58.02 ± 2.68	58.4 ± 1.65$	59.09 ± 2.08
MCH [pg]	base	18.5 ± 0.76	17.94 ± 0.73	17.91 ± 0.69	18.4 ± 0.65
	120 min	18.33 ± 0.86	17.86 ± 0.79	17.59 ± 0.51	17.86 ± 0.85$
	240 min	18.39 ± 0.96	17.79 ± 0.71	17.48 ± 0.61*$	17.91 ± 0.75$
	360 min	18.43 ± 0.9	17.77 ± 0.76$	17.5 ± 0.63*$	17.79 ± 0.99$
MCHC [g/L]	base	306.33 ± 6.81	308.33 ± 4.42	310.44 ± 9.31	312.33 ± 5.3
	120 min	301.92 ± 1.98	304.06 ± 4.75$	302.81 ± 6.92$	301.33 ± 8.87$
	240 min	303 ± 3.86	302.78 ± 4.67$	300.75 ± 6.93$	303.28 ± 6.06$
	360 min	304.5 ± 4.46	303.5 ± 5.28$	299.63 ± 8.62$	300.94 ± 8.77$
Plt [10^9^/L]	base	525.5 ± 107.01	540.19 ± 130.98	537.13 ± 103.67	491.57 ± 50.28
	120 min	500.25 ± 117.98	508.53 ± 92.94	476.64 ± 86.22	426.9 ± 65.29$
	240 min	441.6 ± 98.33	474.94 ± 64.57	469.86 ± 77.03	411.17 ± 71.83$
	360 min	439.3 ± 93.35	508.64 ± 56.15	454.85 ± 61.71	400.4 ± 74.6#$

RBC, red blood cell count; WBC, white blood cell count; Hgb, hemoglobin; Hct, hematocrit; MCV, mean corpuscular volume; MCH, mean corpuscular hemoglobin; MCHC, mean corpuscular hemoglobin concentration; Plt, platelet count. Means ± S.D.; p < 0.05, * vs. Control, # vs. *E. coli* bacteremia, $ vs. base

### 3.2 Red blood cell aggregation

**Figure 1:**
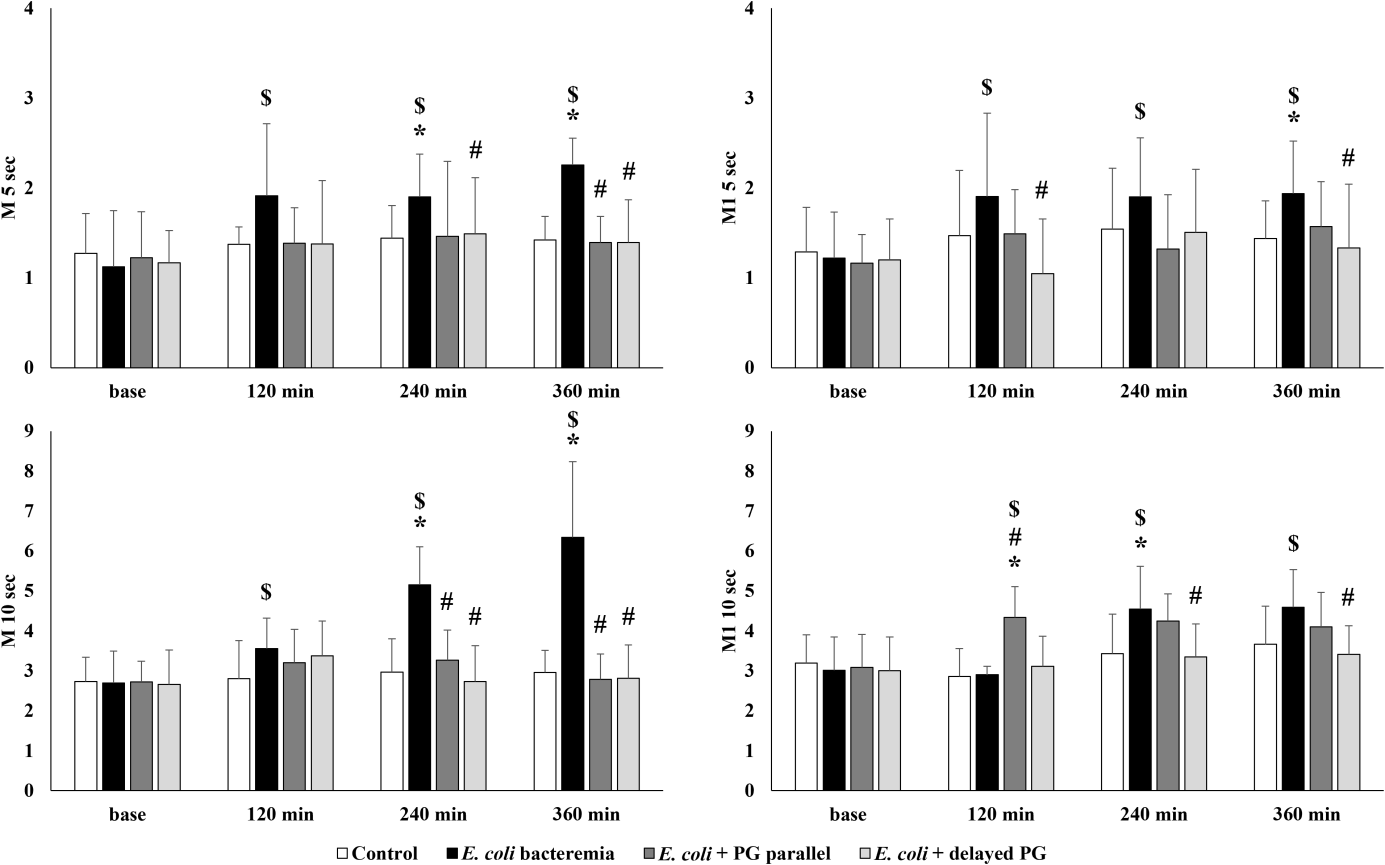
Aggregation index values measured in stasis at 5 or 10 seconds (M 5 sec, M 10 sec) and in 3 s^–1^ at 5 or 10 seconds (M1–5 sec, M1–10 sec) after disaggregation. Means ± S.D.; p < 0.05, * vs. Control, # vs. *E. coli* bacteremia, $ vs. base.

Red blood cell aggregation showed marked differences between groups (**Figure 1**). Aggregation - defined as the reversible linking of cells at low shear rates or stasis - increased in all groups, but to a lesser extent in the *E. coli* + PG parallel, and *E. coli* + delayed PG groups. Larger aggregates and higher shear forces were needed to disperse them in the *E. coli* bacteremia group, while such alterations were absent in the *E. coli* + PG parallel, and *E. coli* + delayed PG groups, suggesting a protective effect of Pentaglobin. In the *E. coli* bacteremia group, we observed significant differences in several measured parameters and time points compared to base values and the values of the Control and *E. coli* + PG parallel, *E. coli* + delayed PG groups. The most marked difference in stasis was observed at 10 seconds of aggregation. In the *E. coli* bacteremia group, the aggregation index values measured at 4^th^ and 6^th^ hours were significantly higher than the values of the Control, *E. coli* + PG parallel and *E. coli* + delayed PG groups (p<0.0001). From the 2^nd^ hour following sepsis induction, these index values also changed significantly compared to base (p=0.0191 vs. 120 min, p=0.0003 vs. 240 min, p=0.0015 vs. 360 min).

### 3.3 Whole blood and plasma viscosity

Whole blood viscosity was measured at various shear rates (**Table 2**). At low shear rates, both *E. coli* bacteremia and *E. coli* + PG parallel, *E. coli* + delayed PG groups showed lower viscosity than Controls at 6 hours. At higher shear rates, the Control and *E. coli* + delayed PG groups exhibited similar values, while the other two groups showed higher ones. At 200 s-¹, viscosity values were nearly identical across all time points.

**Table 2:** Whole blood viscosity, plasma viscosity, and calculated parameters in the Control, *E. coli* bacteremia, *E. coli* + PG parallel, and *E. coli* + delayed PG groups.

Variables		Control	*E. coli* bacteremia	*E. coli* + PG parallel	*E. coli* + delayed PG
WBV at 50 s^-1^ [mPas]	base	3.26 ± 0.59	3.17 ± 0.27	2.49 ± 0.41#	3.04 ± 0.16
	120 min	3.41 ± 0.73	3.02 ± 0.48	2.96 ± 0.3	3.5 ± 0.58
	240 min	3.45 ± 0.45	3.48 ± 0.3	3.51 ± 0.74$	3.41 ± 0.66
	360 min	3.56 ± 0.48	3.19 ± 0.41	3.22 ± 0.52$	3.14 ± 0.45
WBV at 100 s^-1^ [mPas]	base	2.99 ± 0.48	3.01 ± 0.2	2.60 ± 0.29	2.86 ± 0.11
	120 min	3.05 ± 0.56	2.95 ± 0.38	2.72 ± 0.25	2.97 ± 0.26
	240 min	3.02 ± 0.35	3.19 ± 0.25	2.8 ± 0.48	2.89 ± 0.39
	360 min	2.91 ± 0.23	3.1 ± 0.15	3.15 ± 0.31$	2.74 ± 0.12#
WBV at 200 s^-1^ [mPas]	base	2.77 ± 0.37	2.81 ± 0.15	2.35 ± 0.35#	2.63 ± 0.19
	120 min	2.77 ± 0.39	2.7 ± 0.36	2.56 ± 0.21	2.76 ± 0.27
	240 min	2.76 ± 0.22	2.91 ± 0.19	2.82 ± 0.53	2.67 ± 0.35
	360 min	2.84 ± 0.31	2.8 ± 0.16	2.76 ± 0.33$	2.57 ± 0.24
PV [mPas]	base	1.41 ± 0.24	1.30 ± 0.05	1.24 ± 0.1	1.27 ± 0.08
	120 min	1.34 ± 0.21	1.36 ± 0.18	1.36 ± 0.11$	1.41 ± 0.19
	240 min	1.52 ± 0.11	1.37 ± 0.18	1.31 ± 0.1*	1.39 ± 0.2
	360 min	1.45 ± 0.15	1.26 ± 0.13	1.25 ± 0.06	1.38 ± 0.2
WBV_40%_ [mPas]	base	3.18 ± 0.42	3.49 ± 0.29	2.9 ± 0.36	3.58 ± 0.4
	120 min	3.42 ± 0.43	3.43 ± 0.27	3.85 ± 0.33$	4.23 ± 0.4*#
	240 min	3.52 ± 0.4	3.56 ± 0.45	4.53 ± 1.28	3.17 ± 0.4
	360 min	3.64 ± 0.4	3.39 ± 0.39	3.63 ± 0.22	3.85 ± 0.69
Hct/WBC	base	11.41 ± 1.19	10.94 ± 1.12	12.37 ± 0.62	10.84 ± 0.83
	120 min	11.43 ± 1.21	11.65 ± 1.56	9.75 ± 0.61#$	9.2 ± 1*#
	240 min	10.85 ± 1.01	10.78 ± 0.94	9.24 ± 1.52	12.58 ± 2.18
	360 min	10.67 ± 0.93	11.55 ± 1.2	10.72 ± 0.64	10.19 ± 0.34#

WBV, whole blood viscosity; PV, plasma viscosity; WBV40%, WBV corrected for 40% hematocrit; Hct, hematocrit. Means ± S.D.; p < 0.05, * vs. Control, # vs. *E. coli* bacteremia, $ vs. base

Plasma viscosity was lower in the *E. coli* bacteriemia and *E. coli* + PG parallel groups than in the other groups at 6 hours. The hematocrit-to-whole-blood viscosity ratio gradually increased in the *E. coli* bacteremia group, whereas it normalized in the *E. coli* + PG parallel, and *E. coli* + delayed PG groups by 6 hours. Applying the Mátrai formula (viscosity corrected to 40% hematocrit), a mild increase was seen in the *E. coli* + PG parallel, and *E. coli* + delayed PG groups - more pronounced in the *E. coli* + PG parallel group - yet by 6 hours, both treatment groups’ results correlated with the Control values.

### 3.4 Microcirculatory measurements

During the analysis of microcirculatory recordings, we observed significant deterioration in the *E. coli* bacteriemia group: blood flow in the vessels slowed down considerably, complete stasis developed in numerous capillaries, and edematous areas were also observed. In many cases, blood flow ceased completely, large red blood cell aggregates formed in the capillaries, and the flow pattern was markedly heterogeneous, with hyperdynamic flow in some capillaries and complete blockage in others, reflecting the classic nature of sepsis-induced microcirculatory dysfunction. 

**Figure 2:**
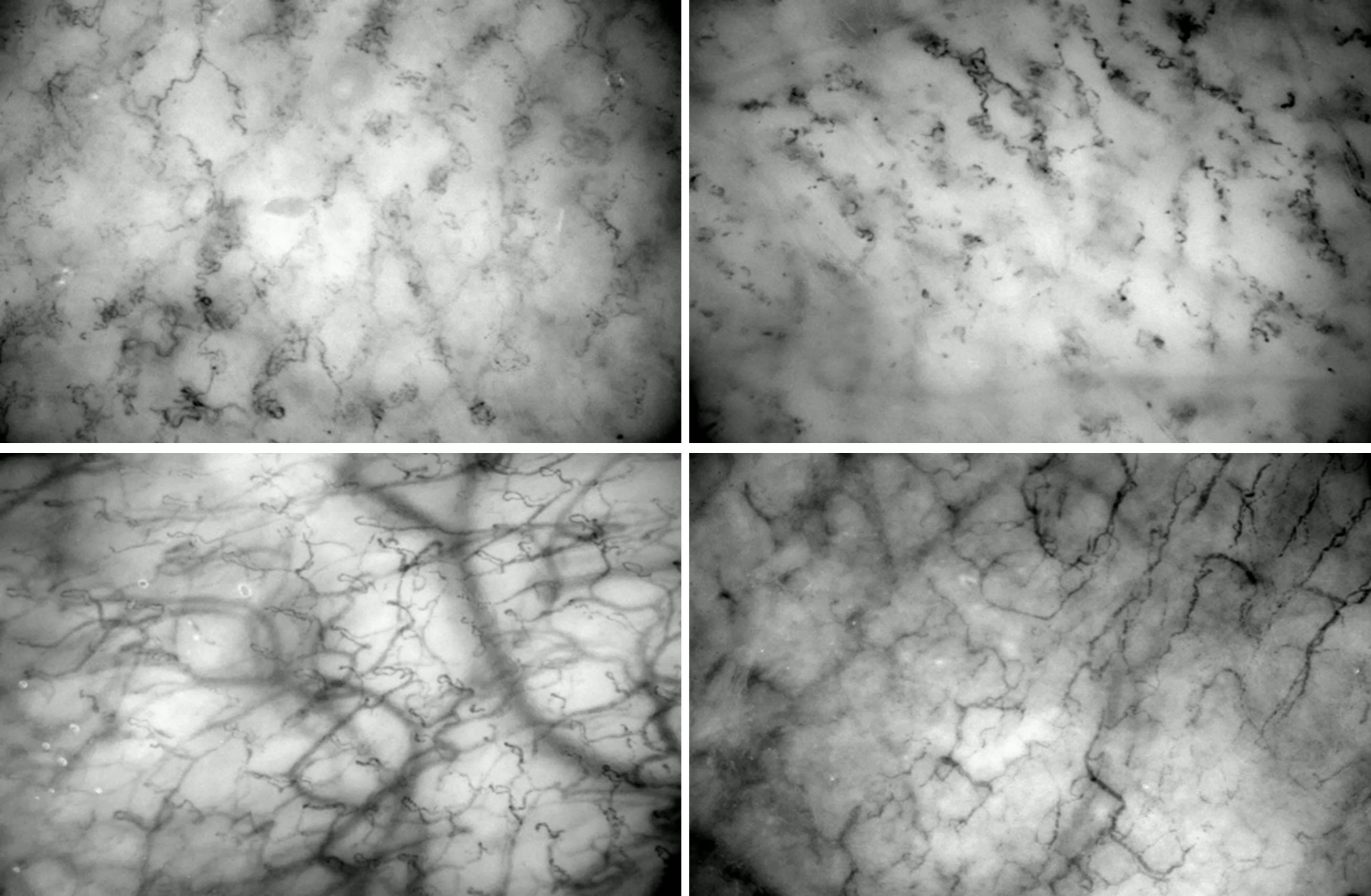
Freeze-frame microcirculation images of the neck skin (above) and sublingual mucosa (below) in the control (left) and *E. coli* bacteremia (right) groups.

In contrast, the *E. coli* + PG parallel, and *E. coli* + delayed PG groups showed improvement. Both forms of treatment resulted in homogenized circulation similar to the flow pattern seen in the control images, significantly reducing stasis and the number of aggregates. These changes are also clearly visible in the images shown in **Figure 2**.

The numerical data obtained during the analysis of the recordings are summarized in **Table 3**. Microcirculatory imaging of the neck skin showed markedly slowed flow and frequent capillary stasis. MFI values declined significantly at 120 minutes before returning toward baseline. De Backer scores and vessel density decreased in the *E. coli* bacteremia and *E. coli* + PG parallel groups, while *E. coli* + delayed PG values remained similar to Controls.

**Table 3:** Microcirculation variables in the Control, *E. coli* bacteremia, *E. coli* + PG parallel, and *E. coli* + delayed PG groups.

Variables		Location	base	120 min	240 min	360 min
MFI [au]	Control	Neck	2.57 ± 0.25	2.54 ± 0.26	2.54 ± 0.33	2.54 ± 0.26
		Ear	2.63 ± 0.53	2.63 ± 0.38	2.50 ± 0.6	2.58 ± 0.36
		Sublingual	2.75 ± 0.26	2.75 ± 0.34	2.71 ± 0.26	2.63 ± 0.31
	*E. coli* bacteremia	Neck	2.52 ± 0.29	2.10 ± 0.19$*	2.09 ± 0.38$*	2.15 ± 0.44$*
		Ear	2.51 ± 0.24	2.39 ± 0.28*	2.36 ± 0.24	2.36 ± 0.21
		Sublingual	2.74 ± 0.27	2.43 ± 0.17$*	2.39 ± 0.18$*	2.34 ± 0.23$*
	*E. coli* + PG parallel	Neck	2.55 ± 0.23	2.50 ± 0.37#	2.50 ± 0.37#	2.47 ± 0.27
		Ear	2.56 ± 0.48	2.47 ± 0.29	2.28 ± 0.31	2.47 ± 0.39
		Sublingual	2.72 ± 0.26	2.53 ± 0.29	2.50 ± 0.37	2.50 ± 0.45
	*E. coli* + delayed PG	Neck	2.58 ± 0.35	2.25 ± 0.26$	2.64 ± 0.38#	2.65 ± 0.23#
		Ear	2.61 ± 0.5	2.22 ± 0.35$	2.36 ± 0.59	2.58 ± 0.52
		Sublingual	2.72 ± 0.34	2.28 ± 0.26$	2.61 ± 0.37#	2.67 ± 0.38#
De-Backer score [1/mm]	Control	Neck	1.22 ± 0.56	1.24 ± 0.28	1.34 ± 0.56	1.37 ± 0.39
		Ear	1.34 ± 0.56	1.23 ± 0.56	1.25 ± 0.4	1.26 ± 0.41
		Sublingual	2.22 ± 0.66	2.08 ± 0.51	2.20 ± 0.58	2.29 ± 0.38
	*E. coli* bacteremia	Neck	1.15 ± 0.31	1.01 ± 0.47	0.97 ± 0.42	1.05 ± 0.12*
		Ear	1.30 ± 0.43	0.88 ± 0.16$*	0.78 ± 0.13$*	0.74 ± 0.26$*
		Sublingual	2.08 ± 0.36	1.18 ± 0.22$*	1.19 ± 0.33$*	1.18 ± 0.2$*
	*E. coli* + PG parallel	Neck	1.14 ± 0.53	1.10 ± 0.18	1.02 ± 0.12	0.98 ± 0.16
		Ear	1.32 ± 0.7	0.89 ± 0.38$	0.92 ± 0.36	0.88 ± 0.17$
		Sublingual	2.18 ± 0.46	1.31 ± 0.35$#	1.34 ± 0.42$	1.53 ± 0.31$
	*E. coli* + delayed PG	Neck	1.18 ± 0.3	0.91 ± 0.47	1.38 ± 0.23$#	1.40 ± 0.1$#
		Ear	1.33 ± 0.44	0.88 ± 0.25$	1.10 ± 0.15#	1.14 ± 0.13#
		Sublingual	2.12 ± 0.45	1.26 ± 0.20$#	1.41 ± 0.25$	1.83 ± 0.31$#
De-Backer vessel density [mm/mm^2^]	Control	Neck	1.07 ± 0.77	1.21 ± 0.4	1.03 ± 0.55	1.38 ± 0.36
		Ear	0.73 ± 0.31	0.63 ± 0.37	0.68 ± 0.17	0.73 ± 0.36
		Sublingual	1.08 ± 0.23	1.03 ± 0.65	0.91 ± 0.47	0.91 ± 0.29
	*E. coli* bacteremia	Neck	1.11 ± 0.12	0.89 ± 0.06$*	0.79 ± 0.09$	0.78 ± 0.09$*
		Ear	0.72 ± 0.05	0.58 ± 0.06$*	0.64 ± 0.02$	0.41 ± 0.05$*
		Sublingual	1.07 ± 0.15	0.80 ± 0.06$*	0.81 ± 0.04$	0.80 ± 0.06$
	*E. coli* + PG parallel	Neck	1.09 ± 0.35	0.89 ± 0.22$	0.91 ± 0.28	0.85 ± 0.23$
		Ear	0.70 ± 0.14	0.58 ± 0.1$	0.72 ± 0.32	0.59 ± 0.44#
		Sublingual	1.02 ± 0.27	0.83 ± 0.23$	0.89 ± 0.33	0.87 ± 0.25$
	*E. coli* + delayed PG	Neck	1.10 ± 0.1	1.05 ± 0.12$#	1.01 ± 0.06$#	1.20 ± 0.15#
		Ear	0.70 ± 0.05	0.65 ± 0.04$#	0.80 ± 0.11$#	0.74 ± 0.05$#
		Sublingual	1.04 ± 0.14	0.96 ± 0.1#	0.97 ± 0.15#	0.99 ± 0.14#

MFI, microvascular flow index, Means ± S.D.; p < 0.05, * vs. Control, # vs. *E. coli* bacteremia, $ vs. base.

In the ear skin, flow was often absent, with pronounced stasis, aggregation, and heterogeneous perfusion. MFI again declined at 120 and 240 minutes. De Backer scores and vessel density were substantially higher in *E. coli* + PG parallel, and *E. coli* + delayed PG groups than in the *E. coli* bacteremia group.

Sublingual recordings showed similar disturbances, with slowed capillary flow and aggregates. MFI changed relative to controls in *E. coli* bacteremia and *E. coli* + PG parallel groups. De Backer scores and vessel density decreased across groups, but Pentaglobin attenuated this decline.

## 4 Discussion

Pentaglobin treatment significantly reduced the increase in whole blood viscosity and red blood cell aggregation caused by experimental fulminant sepsis, changes that may underlie the microcirculatory disturbances characteristic of the model. The post-treatment form (administration following sepsis induction) showed a greater effect on both viscosity and aggregation parameters, presumably due to the better pharmacokinetic profile of the treatment and its immunomodulatory effect in the early phase of sepsis. The pretreatment protocol also showed a positive trend, but to a lesser extent.

As a consequence of fulminant sepsis, pronounced microcirculatory impairment was observed within the capillary networks of both the skin and mucosal surfaces. The alterations included reduced capillary perfusion, heterogeneous flow patterns, and a decline in functional capillary density—all characteristic features of sepsis-induced microvascular dysfunction. These changes reflect the combined impact of inflammatory mediator release, endothelial injury, increased capillary permeability, and microthrombus formation, ultimately leading to compromised tissue oxygenation.

Based on our results, Pentaglobin appeared to mitigate several of these sepsis-related disturbances. In both dosing regimens examined, the treatment was associated with improved microvascular parameters and a partial preservation of tissue perfusion. These findings suggest that Pentaglobin may exert a protective effect on microcirculation, potentially through immunomodulatory actions that reduce endothelial activation and limit further microvascular damage.

Red blood cell aggregation increased during fulminant sepsis, which may be due to elevated plasma fibrinogen levels and changes in red blood cell properties. The acute phase response that develops during sepsis increases the production of fibrinogen, a protein that plays an important role in the formation of rouleaux structures in red blood cells. In addition, sepsis impairs the deformability of red blood cells and increases lipid peroxidation, which also promotes red blood cell aggregation. Research reports that these factors make cells more prone to clumping even in standardized media such as dextran (Baskurt et al., 1997). Increased fibrinogen levels can directly increase red blood cell aggregation, which is associated with the severity of inflammation (Baskurt et al., 1997). Decreased red blood cell deformability and oxidative damage can increase aggregation independently of the effects of plasma (Baskurt et al., 1997; Bateman et al., 2017). In fulminant sepsis, increased aggregation and interactions between platelets and neutrophils can impair tissue perfusion by obstructing microvascular flow (Kirschenbaum et al., 2000). A significant portion of rheological changes occur in the early stages of sepsis and may serve as potential diagnostic markers. Despite apparently adequate circulation, they impede oxygen delivery, promoting the development of organ dysfunction (Kirschenbaum et al., 2000; Ko et al., 2018).

Reduced red blood cell deformability impedes passage through narrow capillaries, which may contribute to microvascular occlusion. In addition, stiffer red blood cells may increase blood viscosity and resistance, which also impedes passage through smaller capillaries (Kim et al., 2015; Ebrahimi and Bagchi, 2022). An apparent increase in blood viscosity and increased vascular resistance may reduce perfusion and cause heterogeneous hematocrit distribution (Ebrahimi and Bagchi, 2022). More rigid red blood cells can prolong the time required for passage through capillaries, reduce functional capillary density, and promote vascular occlusion (Ebrahimi and Bagchi, 2022; Rathnam et al., 2025). These changes may also impair oxygen delivery, thereby exacerbating tissue hypoxia. During sepsis, along with increased aggregation, they may cause microcirculatory dysfunction and organ failure (Kim et al., 2015; Ebrahimi and Bagchi, 2022).

Sepsis rigidifies red blood cells through lipid peroxidation, elevated thiobarbituric acid reactive substances (TBARS), and changes in red blood cell membrane properties (e.g., decreased recovery time), which can be detected even at low shear stress (Baskurt et al., 1998; Vanderelst et al., 2022). Prolonged (days-long) deterioration in deformability indicates a poor prognosis and may promote erythrophagocytosis through reduced complement regulators such as CD35 (Vanderelst et al., 2022). It may contribute to multiorgan dysfunction through increased viscosity and delayed capillary transport (Baskurt et al., 1998).

Pentaglobin treatment has shown promise in the treatment of sepsis. It can inhibit inflammation caused by infection, neutralize bacterial endotoxins and exotoxins, enhance opsonization and phagocytosis, reduce the levels of pro-inflammatory mediators, and enhance the effects of anti-inflammatory mediators (Salman et al., 2020). It regulates the cytokine network, neutralizes activated complement factors, and binds to Fc receptors, thereby limiting excessive inflammation and cell death in immune cells (Domizi et al., 2019). Studies confirm that early administration of Pentaglobin reduces mortality, leads to a more rapid reduction in systemic inflammation, and improves the outcome of sepsis in cases of severe sepsis, particularly sepsis caused by Gram-negative pathogens (such as *E. coli*) (Norrby-Teglundet et al., 2006; Salman et al., 2020; Martinez et al., 2021).

Pentaglobin, an immunoglobulin preparation rich in IgM, can primarily moderate the increase in red blood cell aggregation in sepsis through immunomodulation, as it limits the increase in fibrinogen concentration and the inflammatory mediators that cause changes in red blood cells. Pentaglobin can neutralize endotoxins and excess cytokines, thereby alleviating the acute phase reaction, which plays a role in the increase in plasma fibrinogen levels, one of the main causes of red blood cell rouleaux formation (Domizi et al., 2019; Carlone et al., 2020). In addition, by restoring immune balance and reducing inflammatory mediators, it can indirectly improve the membrane properties of red blood cells, thereby improving oxidative damage and stiffness that promote increased aggregation.

In a single-center, randomized, double-blind, placebo-controlled phase II trial, an IgM-rich infusion (Pentaglobin) at a dose of 5 mL/kg/day administered over 3 days increased microvascular perfusion in the sublingual region (Domizi et al., 2019). A 72-hour infusion of Pentaglobin increased perfusion vessel density (in the small vessels) and the microvascular flow index, whereas these parameters deteriorated in the placebo group. However, the observed improvement occurred without a significant change in mean arterial pressure, and no correlation was found between changes in microvascular perfusion and macrohemodynamic parameters. The study confirmed a decrease in IL-6 and IL-10 levels in the treated group, suggesting that Pentaglobin may alleviate sepsis-induced endothelial dysfunction and glycocalyx degradation, which may underlie microcirculatory disturbances and heterogeneity, by neutralizing endotoxins, regulating cytokines, and binding complement factors. The underlying mechanisms are not yet fully understood, and further research is needed to clarify them.

Clinical studies have shown a reduction in the severity of infection and markers of organ dysfunction, supporting the rheological improvement. These effects occur early at standard dosages, promoting tissue oxygenation (Tugrul et al., 2002; Salman et al., 2020; Nassir et al., 2021). Reduced red blood cell aggregation, along with a decrease in the number and size of blood flow-impeding aggregates, can lower blood viscosity at low shear rates and improve uniform capillary perfusion (Reinhart et al., 2017; Nader et al., 2022).

One milliliter of Pentaglobin solution contains 50 mg of plasma protein, at least 95% of which is immunoglobulin: IgM (6 mg/ml), IgA (6 mg/ml), and IgG (38 mg/ml). Pentaglobin at a concentration of 1 mg/ml is capable of significantly inhibiting lymphocyte proliferation and cytokine release, which may indirectly reduce fibrinogen-mediated red blood cell aggregation, although no direct studies have been conducted in this regard (Nachbaur et al., 1998; Rieben et al., 1999).

Research has reported opsonic activity and reduced platelet aggregation when standard dilutions are used. Higher doses (e.g., 10-fold) of IgG-dominant IVIG analogues show weaker anti-inflammatory effects compared to IgM-rich fractions (Nachbaur et al., 1998). Higher concentrations, such as IVIG containing 10 times more IgG, showed a weaker effect, which may indirectly demonstrate the efficacy of IgM. However, further targeted studies are needed to elucidate the exact process and the relationship between red blood cell aggregation and dosage (Nachbaur et al., 1998; Wand et al., 2016; Lazari et al., 2020; McCulloch et al., 2021).

IgM has a pentameric structure, which allows it to bind with high affinity to bacterial endotoxins (lipopolysaccharides), thereby promoting phagocytosis and clearance without direct interaction with fibrinogen. This can systemically reduce the degree of inflammation and the acute phase response that raises fibrinogen levels, thereby indirectly alleviating red blood cell rouleaux formation (Wand et al., 2016).

Studies have reported lower fibrinogen concentrations following administration of Pentaglobin (e.g., 475 mg/dl compared to 311 mg/dl in the control group), which is due to weakened platelet activation and reduced consumption of endotoxin-induced coagulation, rather than IgM-fibrinogen binding. Currently, there are no structural or affinity studies that could confirm interactions between IgM and fibrinogen D-domains or other sites promoting rouleaux formation (Wand et al., 2016; Reza Hashemian et al., 2018; Schmidt et al., 2021).

Our study has some limitations. As is known, in the intravenously inoculated bacterial infection model, the clinical development is rapid; pathogens introduced into the bloodstream have the greatest immediate impact on the endothelium and vascular system. The consequently developed hypodynamic circulation is accompanied by an increase in serum levels of total cytokines, and without therapeutic intervention, the outcome of the model is fatal. In our study, we did not measure cytokine levels, nor did we investigate the exact mechanism of intravenously administered IgM- and IgA-enriched immunoglobulins; these need to be investigated in the future.

## 5 Conclusion

The model is suitable for further study of Pentaglobin’s protective role. It allows not only the acute effect of treatment to be examined, but also long-term hemorheological changes (e.g., red blood cell deformability) and the testing of new therapeutic combinations. Our results support the potential clinical applicability of Pentaglobin in the treatment of sepsis-associated microcirculatory disorders.

Despite these promising observations, the optimal timing (whether therapy should be initiated before or after the onset of sepsis) for Pentaglobin therapy remain to be fully defined. Additional experimental and clinical studies are therefore required to clarify its mechanisms of action, determine its efficacy across different stages of sepsis, and refine its integration into standardized treatment protocols.

## Data Availability

The original contributions presented in the study are included in the article/supplementary material. Further inquiries can be directed to the corresponding author.
